# Non-Invasive Raman Classification Comparison with pXRF of Monochrome and Related Qing Porcelains: Lead-Rich-, Lead-Poor-, and Alkali-Based Glazes

**DOI:** 10.3390/ma17143566

**Published:** 2024-07-18

**Authors:** Philippe Colomban, Xavier Gallet, Gulsu Simsek Franci, Nicolas Fournery, Béatrice Quette

**Affiliations:** 1 Sorbonne Université, CNRS, MONARIS UMR 8233, Campus P. et M. Curie, 4 Place Jussieu, 75005 Paris, France; 2 Musée National d’Histoire Naturelle, CNRS, Université Perpignan Via Domitia, Musée de l’Homme, UMR 7194—Histoire Naturelle de l’Homme Préhistorique (HNHP), 17 Place du Trocadéro, 75116 Paris, France; xavier.gallet@mnhn.fr; 3 Koç University Surface Science and Technology Center (KUYTAM), Rumelifeneri Yolu, 34450 Istanbul, Türkiye; gusimsek@ku.edu.tr; 4 Galerie Nicolas Fournery, 75001 Paris, France; nf@galerienicolasfournery.fr; 5 Musée des Arts Décoratifs, 111 Rue de Rivoli, 75001 Paris, France; beatrice.quette@madparis.fr

**Keywords:** China, glaze, monochrome, porcelain, composition, Qing Dynasty, Raman, pXRF, non-invasive classification, Pb content

## Abstract

Chinese porcelain with an optically clear colored glaze, imported to Europe from the Kangxi period (1662–1722, Qing Dynasty) onwards was highly collected by the French Elite of the 18th century. The bright colors with a clear, shiny glaze were unlike anything produced in Europe at that time. The colors of enamelled artifacts (on biscuits or already glazed porcelain) can be fully monochrome or consist of associations of large monochromatic areas with or without application of gilding. Non-invasive portable XRF and mobile Raman analyses have previously shown their effectiveness in the characterization of (colored) glassy silicates. In this study, we compare the Raman signatures of twenty-one Chinese artifacts fully—or with major monochrome area (*sancai*)—decorated with blue, turquoise (or celectian blue), honey-yellow, green, eggplant, and red color. Different types of glazes are identified and confirmed by pXRF: lead-rich, lead-poor-alkali, lead-doped alkali, and alkali-based compositions. However, an unexpected low level of lead is observed in the turquoise glazes, likely to optimize the gloss. Raman spectroscopy appears more reliable to compare the Pb content than pXRF. This work presents Raman spectral signatures of glazes that can potentially be used for non-invasive object classification and counterfeit detection.

## 1. Introduction

The variety of categories of ceramics, mainly Chinese porcelain, has been well-established for a long time [1]. Numerous works analyzing the compositions of the pastes have been published since the pioneering work of famous scientists Ferchault de Réaumur at the beginning of the 18th century [2,3,4], then Brongniart [5] in the 19th century, and many others. The series of books edited by Kingery [6,7,8,9], with the contribution of Li in particular [10] in the 1980s, provided a first understanding of the pastes and their preparation within a modern view. Studies concerning the nature of coloring agents and their vitreous silicate matrix, called glaze (fired with the body) or enamel (or overglaze, deposited and fired on the already glazed/fired body), are more limited and recently performed. We can cite the pioneer works of Zhang [11] and then Wood [12] about the productions of the Ming, Yuan, and earlier dynasties. Indeed, for a long time, a certain quantity of material was needed to analyze enamels, and the process was macro- or micro-destructive, hence limiting most of the studies to shard analyses only. Sampling a body fragment with the required matter volume was relatively straightforward, even on an ‘intact’ artifact. In contrast, sampling the same volume of glaze or enamel, namely a layer between 0.1 and 1 mm of thickness, was more complex and impossible for rare masterpieces. The development of non-invasive, mobile analytical techniques, namely X-ray fluorescence (XRF) [13] and Raman spectrometry [14], offers an alternative.

White, blue-and-white, and celadon wares are the most famous Chinese ceramics and receive much attention (e.g., refs. [15,16,17,18,19,20,21]). Their glazes are relatively thick. Shipwreck and kiln site excavations provide many shards and broken pieces, which enable a micro-destructive comprehensive analysis with possible dating. Most of the works deal with the elemental composition analysis on such shards. 

Chinese artifacts were first imported to Europe during the last period of the Ming Dynasty by Portuguese merchants [22,23]. The volume of trade drastically increased when Dutch and English and, to a smaller extent, French and Swedish traders dominated the exchange during the 18th century, i.e., during the Qing Dynasty [24,25,26,27,28,29,30,31]. The bright color with a clear, shiny glaze of some production was unlike anything manufactured in Europe then. In China, turquoise blue glaze was initially produced for the decoration of architectural works (Yaozhou kiln, Tang dynasty). The turquoise blue color began to be used in ceramics during the Song dynasty (960–1279) and was then widely used in the northern Chinese ceramic factories during the Jin (1115–1234) and Yuan dynasties (1279–1368), on coarse “Cizhou” stonewares [1]. In the 14th century, the Mongol invasion from the north led to the migration of most artisans to the south, where the production technique of turquoise blue spread, particularly in Jingdezhen. The turquoise blue color was known in ancient China as *cuilan* (翠蓝), and is also often referred to as *facui* (法翠) due to its use in Fahua (法华) ceramics in the Shanxi region. Nowadays, it is more commonly known as “peacock blue”. Rare turquoise-glazed porcelains with the Xuande mark (1425–1435) are conserved at the Palace Museum Taiwan and British Museum, and were expected to be made in Jingdezhen.

The simplicity and brilliance of the colors, allied either with the purity of forms inherited from the Song Dynasty or, in contrast, with the exoticism of figurine shapes, were trendy in France during the 18th century, and to this day, many pieces can be found in French private and public collections. The French elites of the 18th century appreciated bright and cheerful colors, as evidenced in the textiles, wallpapers, and pastries of this period. During the 18th century, French authorities endeavored to promote porcelain production by assaying to collect technical information on Chinese hard-paste processes [32,33]. Conversely, the Chinese authorities requested the help of the Jesuit missionaries settled at the Manchu Court to supervise the establishment of imperial glass and enameling workshops, which are capable of reproducing polychromatic painted enameled objects similar to those imported from Europe [34,35,36,37,38,39]. 

Monochrome glazes are colored by the ‘simple’ dissolution of transition metal ions (Fe: yellow; Mn: eggplant; Co: blue; Cu: green to turquoise) into the amorphous silicate network and are called ‘*couleurs transparentes*’ (in English: *optically clear color*) [12,40,41]. High concentrations of coloring ions lead to saturation and precipitation of specific phases with a loss of transparency and sometimes of brilliance. Achievement of yellow, eggplant, blue, green, and turquoise colors is well established [11,12]. The composition of the glaze (lead-based or alkaline) can modify the hue, e.g., jade green (lead-based glaze) or turquoise (alkaline glaze) for Cu-containing glaze [11,12,40,41]. Therefore, if these coloring elements do not lead to phase precipitation, the Raman spectrum will be similar to that of the uncolored silicate matrix and the technique will only be efficient to identify the type of glassy matrix [14]. The continuous fluorescence background can still be affected, either due to impurities leading to a specific emission (chromium and rare earth) and broad features because of the electronic defects (e.g., substitution with manganese) [41,42,43,44]) or due to the absorption of partial scattered radiation; the matter also flattens the background differently as a function of its absorption of color. 

Previous comprehensive Raman studies mainly concern Chinese and Vietnamese celadons [45,46,47,48], the colorless glazes of Yuan and Ming porcelain [17,21], and painted enamels of the Qing Dynasty [37,38,39,49,50]. To our knowledge, no systematic combined XRF and Raman non-invasive study of monochrome china, particularly using mobile instruments, has been published. We report here the analysis of a selection of 18th-century Qing porcelain fully glazed or decorated with large monochrome areas. Usual descriptions assume that the glaze was made on a ‘biscuit’, i.e., directly applied on the porcelain body previously fired at high temperature without any colorless glazing. We will discuss their glazing routes and compare measurements made at the laboratory with bench-top ‘big’ instruments with those performed with a mobile Raman set-up.

## 2. Method and Artifacts

### 2.1. Artifacts

The twenty-one studied artifacts are listed in Table 1. They are stylistically assigned to the Qing Dynasty, from Kangxi (1661–1722) to Qianlong (1735–1796) reign. Twelve figurines and one cup, mainly from Kangxi reign, belonging to a private collection, were analyzed at the laboratory with Raman HR 800 and/or portable XRF spectrometer. Five bowls, a Budai maggot, and a bottle of the Musée des Arts Décoratifs (Paris) collection [51] were analyzed with mobile Raman HE532 and/or pXRF spectrometers in the storage rooms.

The selected objects are representative of artifacts imported in France during 18th and 19th centuries.

#### Description of the Artifacts

**Figure 1:**
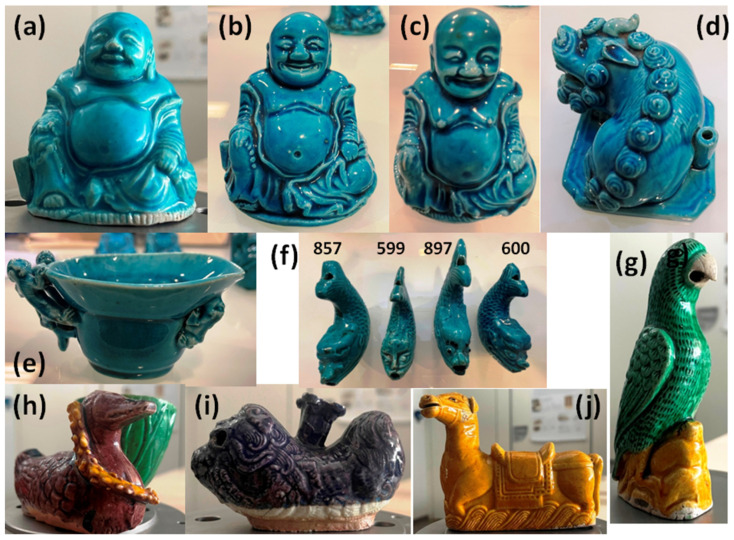
Monochrome and tricolored (*sancai*) figurines: (**a**) Budai 0, (**b**) Budai 1, (**c**) Budai 2, (**d**) lion, (**e**) cup, (**f**) fishes (inventory numbers are given in Table 1), (**g**) parrot, (**h**) duck, (**i**) lion (*fo* dog), and (**j**) yellow horse (private collection).

**Figure 2:**
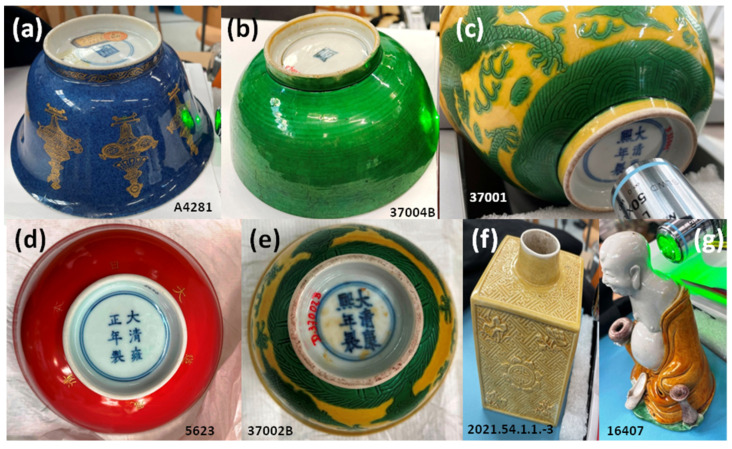
Monochrome and tricolored artifacts (Musée des Arts décoratifs Collection); inventory number are given (see Table 1).

*Fo dog/Lion* (Figure 1d,i)*:* The mythic version of the animal, also known as the dog of *fo* (or foo), derives its name from the Chinese word ‘*fo*’, which often refers to a monk identified with Maitreya Buddha. The Buddhist version of the dog was originally introduced to Han China (ca. 200 BCE) as the protector of dharma. Gradually, they were incorporated as guardians of the Chinese Imperial Law. However, Chinese sensitivity metamorphosed the dog into a lion, despite lions not being indigenous to China, as this seemed more fitting to the dignity of an emperor when he used the beasts to guard his gates. The mythic dog is also associated with *feng shui* and is often called *fu* dog in Western texts (*fu* means ‘happiness’ in Chinese). They are also called *rui shi* (‘auspicious lion’) or simply *shi* (lion). One lion (Figure 2d) is turquoise, and the second (Figure 2i) is eggplant color.

*Parrot* (Figure 1g)*:* Green and yellow glazes are associated with this piece. Parrots are favorite birds of the bodhisattva Guanyin. In China, parrots are found in the wild in the warmer southern provinces. Since at least the Tang dynasty (618–907), exotic birds from Indochina and Indonesia were brought to the imperial court. During Kangxi’s reign, parrots depicted in various porcelain colors were popular in the West due to their vibrant and exotic appearance. In Europe, the taste for parrots was further encouraged by the fashion of exotic orientalism when ownership of live parrots was also widespread. Models of small parrots were found in the VOC wreck.

*Mandarin duck* (Figure 1h): The theme of mandarin ducks is common in porcelain Chinese décor. Yellow, eggplant, and green colors are used.

*Sitting yellow horse* (Figure 1j): The horse is shown with its tack, including stirrups, bridles, halters, reins, bits, and harnesses.

*Budai* (*fo*) (Figure 1a–c): The turquoise symbol of happiness, modeled in a seated pose, wearing a loose robe, leaving his belly exposed, and holding a necklace of pearls in the left hand decorated in turquoise enamels on the biscuit, with a cylindrical incense holder on the side. Budai is a Chinese deity. His name comes from the bag he carries. According to Chinese tradition, Budai was an eccentric Chinese monk who lived in the 10th century. He almost always appears smiling or laughing, hence his nickname in Chinese, the “*Laughing Buddha*”. In English-speaking countries, he is also known as the “*Fat Buddha*”. Budai is often depicted as a bald, generously proportioned man dressed in a robe and wearing prayer beads. He carries his few belongings in a canvas bag. His figure appears in Chinese culture as a representation of contentment. Budai figurines often have a thin stick of incense burning on the side of the spout provided. They were probably used in family shrines accompanying prayers, but were considered exotic curiosities in the West. It could also be placed on the desk of a Chinese scholar to measure the time when the incense stick was consumed. Some figures were used in different forms, such as brush washers. Budai figures were sometimes called Maggot or Pagoda.

*Fishes* (Figure 1f): Turquoise glazed water droppers are modeled as mythical fish. The Chinese word for fish, ‘*yu*’, is pronounced like the word for abundance. So, fish have come to represent prosperity.

The five bowls studied with mRaman set-up are attributed to the Kangxi, Yongzheng, and Qianlong reigns [51]. Yellow-colored bowls, highly appreciated by the Qing Court, are decorated with the imperial dragon (Figure 2c,e). Typical blue (Figure 2a) and red (Figure 2d) bowls from the Qing Dynasty are included in the group to compare with standard high-temperature glazed porcelain. Incised yellow-green bowls belong to a group decorated with incised dragons in green or blue. The Budai maggot (Figure 2g) and the bottle (Figure 2f) complete the variety of hues for yellow.

### 2.2. Spectroscopic Analysis

#### 2.2.1. Elemental Composition

The measurement and data evaluation procedures have been described in detail in previous articles [38,39]. X-ray fluorescence analysis was performed on site using a portable ELIO instrument (Milano, Italy). The set-up included a miniature X-ray tube system with a Rh anode, a ~1 mm^2^ collimator, and a large-area Silicon Drift Detector with an energy resolution of <140 eV for Mn Kα, covering an energy range (in air) from 1.3 keV to 43 keV. The working distance was 1.4 cm. 

We measured the diorite D-RN geological standard provided by the ANRT (*Association Nationale de la Recherche Technique*) in the form of a polished rock piece to control the stability of the instrument’s performance. Perfect perpendicularity to the area measured was maintained. Measurements were performed in point mode with an acquisition time of 150 s, using a tube voltage of 50 kV and a current of 80 μA. No filter was interposed between the X-ray tube and the sample. The analysis depth during enamel measurement was estimated using the Beer–Lambert law (analysis depth, defined as the thickness of the top layer from which comes 90% of the fluorescence can be calculated for each characteristic X-ray peak using the online calculation program provided by Bruker [52]) to be close to 6 µm at Si Kα, 170 µm at Cu Kα, 280 µm at Pb Lα, 300 µm at Au Lα, and 2.5 mm at Sn Kα.

The data fitting procedure has already been explained in previous papers [38,39]. After recording the raw data, the spectra files were opened using Artax 7.4.0.0 (Bruker, AXS GmbH, Karlsruhe, Germany) software. For the data treatment process, the studied objects were considered as infinitely thick samples. The net area was calculated under the peak at the characteristic energy of each element selected from the periodic table, and the counts of major, minor, and trace elements were determined in the colored areas. Before plotting the scatter diagrams, the net areas of each element were normalized by the number of XRF photons obtained from the elastic peak of the rhodium X-ray tube. These normalized data were then plotted in ternary scatter plots, which were used for the interpretation and discussion of the results with the software Statistica^®^ 13.5.0.17 (TIBCO Software Inc., Palo Alto, CA, USA). For interpretating the results with a statistical approach, a hierarchical Euclidian clustering diagram was created by using data obtained from the XRF photons of Pb, K, Mn, and As, also utilizing Statistica^®^. Assuming overall homogeneity and infinite thickness (correct for the paste, nearly correct for areas where enamel is thick), compositions were evaluated using the software provided with the instrument. 

#### 2.2.2. Phase Identification

Raman spectra were recorded using two Raman set-ups: (i) in the laboratory, with a Labram HR800 spectrometer (HORIBA Scientific Jobin-Yvon, Longjumeau, France) excited by an Ar^+^ ion plasma laser Innova I90C 6UV (Coherent Inc., Saxonburg, PA, USA). The 457.9 nm line was used with approximately 2 mW power of illumination on the sample surface analyzed by using long working distance objectives (LWD) of 50× and 100× (Olympus Corp., Tokyo, Japan); or, (ii) an HE532 mobile spectrometer (HORIBA Scientific Jobin-Yvon, Palaiseau, France) excited with a 532 nm YAG laser Ventus (Laser Quantum, Fremont, CA, USA) with power of illumination ranging between 5 and 20 mW, fiber optic coupled with a remote Superhead^®^ equipped with a 50× (Nikon, Tokyo, Japan) or a 200× (Mitutoyo, Sakado, Japan) magnification microscope objective. The 600 and 900 g/mm gratings were, respectively, used in order to record a spectrum with a large spectral range (~100–3300 (HE532)/100–3900 (HR800) cm^−1^, with three windows for HR800 measurements). Analyzed spots are smaller than ~5 × 5, 2 × 2, and 1 × 1 µm^2^, respectively; the in-depth penetration is rather similar for the colorless and poorly colored glazes, but about 5–10 times smaller for darker colored areas. It is well established that blue (or violet) laser excitation is the most efficient way to record the Raman fingerprint of silicates. Three to five spectra were recorded for each colored area to ensure representativeness.

## 3. Results

### 3.1. Compositions and Coloring Agents of Monochrome Glazes

We will first summarize previously published results regarding monochrome glazes.

#### 3.1.1. Colors

Yellow is the Chinese Imperial color. Imperial yellow, especially brilliant, is based on iron (Fe^3+^) ions added as coloring agent in lead-rich glaze [11,12,53,54] and not on the addition of pyrochlore tin-rich or antimony-rich pigment as used in painted opaque enamel. These two later pigments are expected to be imported from Europe during the Wanli (end of Ming Dynasty, perhaps due to the introduction of this pigment in Arita (Japan) by Portuguese missionaries in circa 1580 [55]) and the Kangxi (beginning of Qing Dynasty) reign, respectively [37,39,49,50,56,57].

Eggplant (formerly called “*violet*” in French and “brownish purple” in 17th and 18th century inventories and now “*aubergine*”) is achieved by dissolving manganese ions (Mn^4+^) in the glassy silicate matrix [11,12,53]. 

Red colors can be obtained using a dispersion of hematite pigment (α-Fe_2−x_M_x_O_3_) [56,57] or copper metal (Cu°) nanoparticles [49,57,58,59] in the glassy matrix.

Green is achieved by dissolving copper (Cu^2+^) ions in the glassy matrix [11,12,53,57,58,59].

#### 3.1.2. Compositions

Table 2 summarizes compositions already published in the literature (see e.g., [60,61,62,63]). 

Comparison of compositional data from the literature for predominantly monochrome porcelains in yellow, red, and green highlights three groups of compositions:

- Compositions very rich in lead, ranging between 55 to 60 wt% PbO;
- Compositions containing a high level of lead, between 40 to 50 wt% PbO;
- Compositions containing average-to-low lead levels, approximatively 20% wt PbO.

A large variability in lead content is observed in the literature [60,61,62,63]. This may result from the variability in the glaze composition deposited on the surface of the body (already glazed or not (i.e., biscuit)), the firing conditions (PbO is very reactive and volatile above 800 °C which will contaminate the entire surface of the artifact decorated with lead-based enamel or all items fired in a kiln previously used to fire objects decorated with lead-based glaze), and the measurement conditions: if the analyzed volume and depth probe is too large, higher than the thickness of the enamel, the substrate initially free of lead will contribute to the measurement and lower the calculated content. We must therefore consider types of glaze and not precise compositions.

### 3.2. XRF Analyses of Figurines

**Figure 3:**
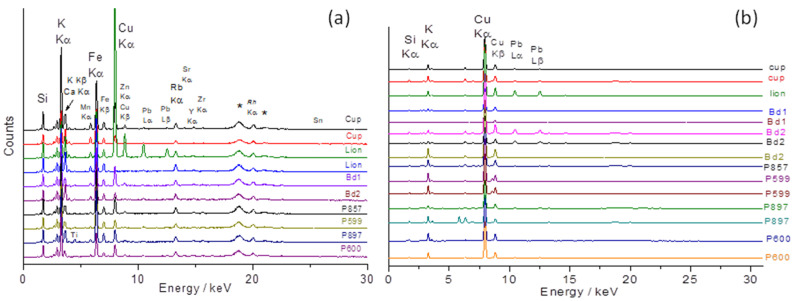
Representative XRF spectra recorded on body (**a**) and glaze (**b**) layers of turquoise-colored artifacts shown in Figure 1. See the text for the artifact codes (* Rh anode peak).

Figure 3 presents the portable XRF spectra obtained on the porcelain paste and the glaze of the objects presented in Figure 1.

**Figure 4:**
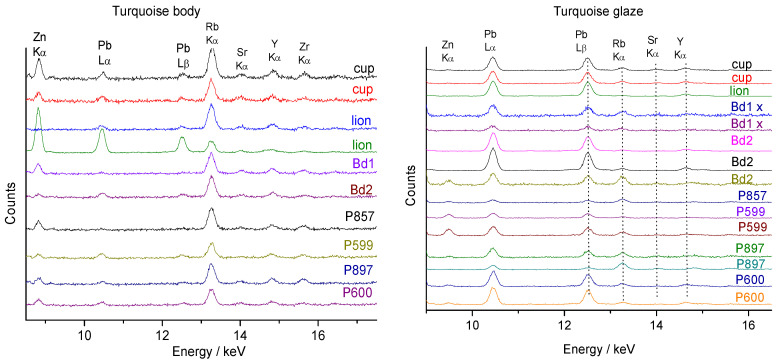
Zoomed-in spectral views on XRF spectra of Figure 3, recorded on body (**left**) and glaze layers (**right**) of turquoise-colored artifacts.

The zoom view on the ~8–17 keV energy range (Figure 4) where some trace elements, namely rubidium (Kα 13.39 keV, impurity associated with sodium and potassium), strontium (Kα: 14.16 keV, impurity associated with calcium), yttrium (Kα 14.96 keV, impurity of sand), and zirconium (Kα: 15,77 keV, impurity of sand) exhibit rather strong XRF signals whatever their low amounts.

**Figure 5:**
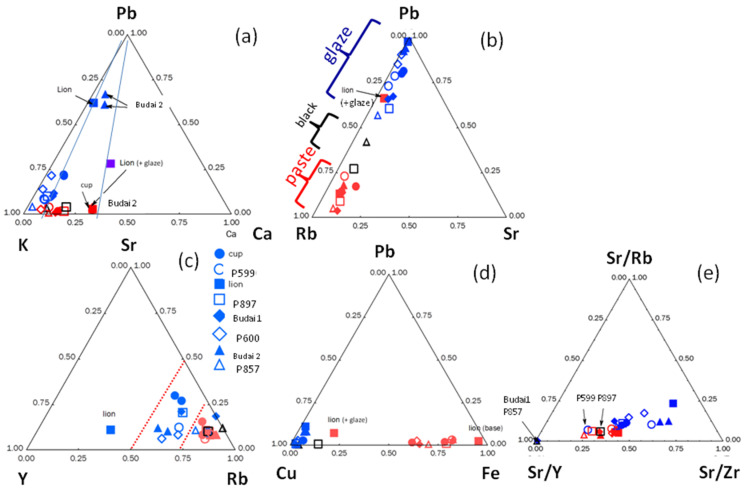
Diagrams made from the relative intensity of the peak characteristic of the elements for artifacts shown in Figure 1: (**a**) fluxing elements (Pb, K, and Ca); (**b**) Pb vs. Rb (impurity of K and Na) and Sr (impurity of Ca flux) signal; (**c**) characteristic impurities of the fluxes (Rb, Sr) and the sand (Y); (**d**) Cu and Fe coloring ions vs. Pb signal; (**e**) Sr proportional to Rb, Y, and Zr signals (see Table 1 for sample number).

Previous studies have demonstrated that the relative intensity of these impurity peaks can be used as a fingerprint of the geological context of raw materials [37,38,39]. Figure 5 compares the ternary diagrams constructed from the areas of the characteristic peaks of the fluxing elements (Pb, K, and Ca; the measurement of Na, Li, and B is not possible with portable X-ray fluorescence spectroscopy, hereafter called pXRF) and compositions evaluated by using the instrument software and given in Table 3. 

The low signal of Pb is a surprise. Indeed, if turquoise color requires the application of an alkaline-based glaze matrix, previous studies [61] show high Pb content (Table 1). The visual comparison of the peak intensity of the M and L transition of lead element with respect to those of the potassium M and K transition, however, indicates a low level of lead. Glazes belong to an alkali-based group, and this low-level addition of lead was not made in order to significantly soften the melting temperature but probably to adjust viscosity and wetting properties. However, adding lead (and boron not detectable by pXRF) is known to increase the gloss [40]. Alternatively, the contamination of the glaze with lead can result from using a kiln polluted by the previous firing of artifacts glazed with lead-based glaze.

The compositions listed in Table 3 suppose that the glaze is homogeneous over a thickness larger than the X-ray beam penetration. The values measured for light elements cannot be thus considered as reliable. The high gloss of the glaze guaranties the absence of corrosion. The low content of iron oxide in the paste is consistent with a porcelain body composition.

The proportions relating to transition metals for which the depth over which the measurement is carried out are comparable to the thickness of the glaze and can be also considered reliable. Usually, ~0.2 wt% of CuO is required to color a glass. The contents of 0.05 to 0.1 wt% CuO measured for Budai 1 and Budai 2 turquoise glaze indicate the contribution of the paste and the glaze of thickness less than 200–250 µm at the measured spot.

Figure 3 shows the maximum intensities of copper for the glaze and potassium and iron for the paste (in one case the Cu peak measured on the ‘body’ surface is important due to a thin burr in the enamel on the paste). Indeed, the contamination of the paste surface with lead is observed in some places for the lion figurine (Figure 3 and Figure 5). Remember that the intensities refer firstly to a function of the electronic transition (K, L, or M lines) specific to the elements considered and secondly to the content. The glazes of fish figurines P857, P599, and P897 appear visually (almost) free of—or with only traces of—lead, while the others contain lead at low levels. Calculation from pXRF measurement gives an estimation of the Pb content for Lion P984, Budai 1, and Budai 2 (Table 3). We will further compare with the Pb content empirically calculated from Raman data. The true Pb content is certainly higher than that measured by pXRF because the in-depth penetration of the beam is deeper than the glaze thickness and hence the contribution of the substrate—the paste free of lead—decreases the calculated value. The dispersion of the glaze data relative to Pb intensity in Figure 5b is assigned to the variable contribution of the paste due to the variable thickness of the glaze. We will see that the level of lead is lower than those mentioned in Table 2 and these glazes cannot described as lead-based but as lead-doped. The pXRF measured content ranges between 0.9 and 2.5 wt% PbO. The examination of the signals of the impurity characteristics of the raw materials, Y, Sr, Rb, and Zr (Figure 5) shows that the pastes of these objects use similar or identical raw materials, which are compatible with the productions coming from the same kiln, except for Budai 2 paste (with more Ca) perhaps. In Figure 5, we can distinguish three different glazes: the lion (more Y), the cup (more Sr), and the others. This could indicate different glazing workshops or different glazing qualities.

The glazes of the turquoise-colored Budai 2 and the lion appear to be the richest in lead among the objects analyzed with pXRF. This is confirmed by the Pb-K-Ca peak intensity diagram in Figure 5a. Note that the comparison of the relative peak area would give values located at or very close to the Pb axis for lead-rich glaze and that the location in the ternary diagram of peak area depends on the selected elements. A surface measurement of the paste of lion figurine contaminated by the volatilization–condensation of lead oxide during firing is clearly apart. The special character of the lion figurine is clear. The lion’s body also has the richest amount of iron. The difference in raw materials used for bodies and glazes is also evident by considering the ternary diagram built from the signal of Sr-Y-Rb impurities (Figure 5b–e). The special character of the lion and Budai 2 glaze is clear whatever criterium is considered.

### 3.3. XRF Analyze of Bols

**Figure 6:**
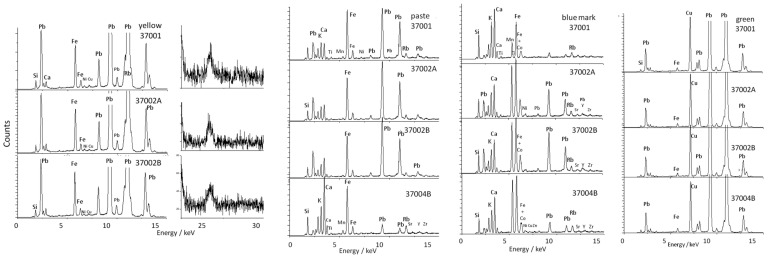
Comparison of the pXRF spectra recorded for bowls of Figure 2 on the paste, the blue mark, and the yellow and green areas. Inventory numbers are given; see Table 1 for description.

**Figure 7:**
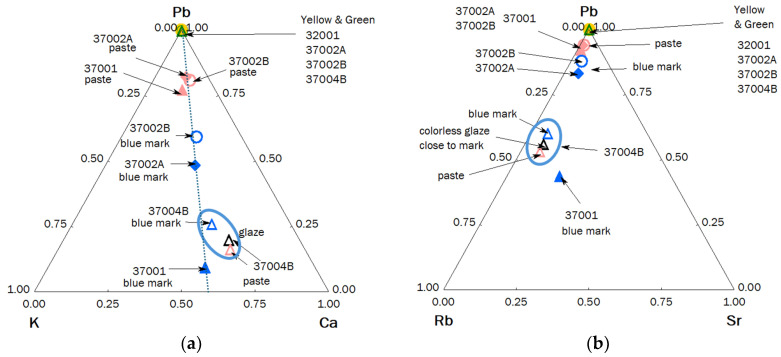

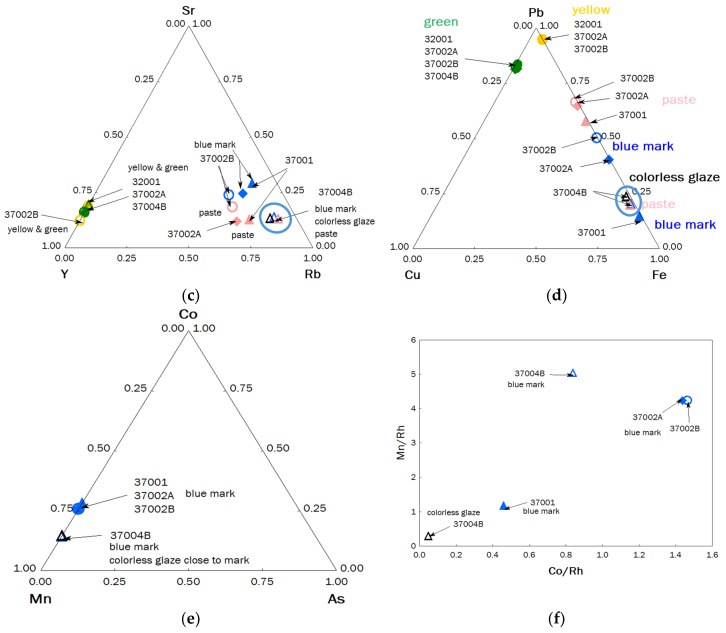
Diagrams made from the relative intensity of the peak characteristic of the elements for some artifacts shown in Figure 2: (**a**) fluxing elements (Pb, K, and Ca); (**b**) Rb, impurity of K and Sr, impurity of Ca flux vs. Pb signal; (**c**) characteristic impurities of the flux (Rb, Sr) and the sand (Y); (**d**) Cu and Fe coloring ions vs. Pb content; (**e**) Co, Mn, and As diagram; (**f**) Mn-Co biplot (intensity normalized with Rh anode peak); inventory number, see Table 1.

Figure 6 compares the pXRF spectra obtained on the paste and yellow and green-colored glazes as well as on the reign marks inscribed in the blue color of the bowl back foot presented in Figure 2. With the exception of bowl 37004B where the intensities of the peaks of the Pb L transitions are very weak (3.6 wt% PbO in Table 3), like those observed for the figurines in Figure 3 and Figure 4, the intensities are significant. Consequently, the glazes of these bowls are rich in lead as shown in Figure 7, which is confirmed by the PbO content measured around 45 wt% (Table 3), a value consistent with the literature (Table 2).

The very low pollution of the 37004B paste surface (rim foot) and 37001 foot center (blue underglaze mark, Figure 6) undoubtedly results from the protection provided by the stacking of the bowls during firing.

This is an indirect proof of the quality of the production. Examination of the intensities of the peaks of the metals associated with cobalt shows that the two small 37002A and 37002B bowls decorated with dragons and the green 37004B bowl were marked with the same source of cobalt, rich in manganese, traditionally used since the Ming Dynasty [37,38], with traces of nickelm significantly. The Kangxi mark of large, yellow-and-green-colored bowl 37001 is different, with a lower level of manganese, which may indicate a mixture of traditional Asian (Ming) and European cobalt used for sophisticated imperial productions [37,38,39].

The yellow and green colors are obtained classically with Fe^2+^ and Cu^2+^ ions as coloring agents. As indicated by the observations of the corresponding Kα and Kβ peaks of these elements (at ~25 and 28.5 keV), a certain level of tin is detected for the yellow glaze (Figure 6). This can be related to some addition of lead–tin pigment, which could be a confirmation of a special production.

Figure 7 compares the relative intensities of the peaks of the elements Rb, Sr, Y, and Zr, with the first two elements characteristic of the fluxes used and the last two of the sands.

The Pb-K-Ca diagram (Figure 7a) shows a constancy in the ratio of the intensities of the peaks concerning the elements K and Ca, a ratio fixed by the composition of the layer below lead-based glaze. The position along the line going towards the apex Pb is a function of the thickness of the enamel and the thickness contaminated during firing. The sublayer, more calcic, is different from that of the figurines (Figure 5). Due to the higher Ca content, it can be assumed that for those artifacts of Figure 2 the lead-based glaze has been made over an already glazed porcelain. In contrast, the glazes of figurines shown in Figure 1 (except Budai 2 and lion Figure 1i) have been made directly on the paste (and for this reason the name ‘biscuit’ is correct). For the lion (Figure 1i), the underglaze is visible. The blue ellipse gives an illustration of the variability in the measurement due to inaccuracies, the variable thickness of the glaze, and the heterogeneity of the material. The Pb-Rb-Sr diagram (Figure 7b), like the equivalent diagram for figurines (Figure 5b), distinguishes glazes containing lead from lightly polluted glazes based on the rubidium signal provided by potassium and sodium. The comparison of the peaks of the impurities of the sand (Y) and the fluxes (Sr and Rb) (Figure 7c) confirms the great similarity of the raw materials used, on the one hand for the glaze yellow and green (the dispersion lower than the variability of the measurements is not significant), and on the other hand the colorless glaze, colored or not by the mark’s cobalt. The green coloration by copper and yellow by iron in a glass very rich in lead are visualized in the diagram Figure 7b. As in Figure 5c, the Sr/Y/Rb intensity ratios separate the glazes from the pastes quite well.

The cobalt used is rich in manganese and arsenic-free (Figure 7c) in accordance with the use of traditional cobalt in Jingdezhen kilns [57]. The content of manganese in the bowl mark 37004B appears the richest.

### 3.4. Raman Categorization

**Figure 8:**
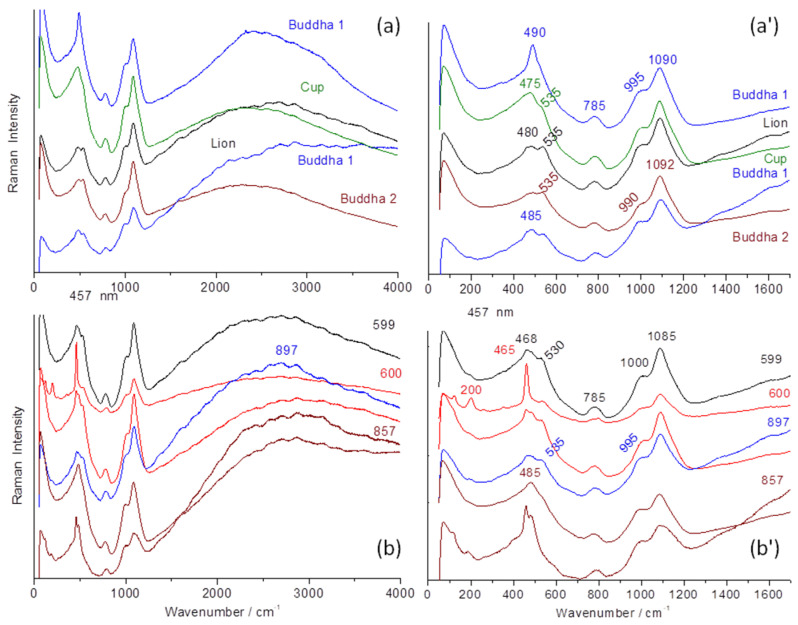
Comparison of the Raman spectra recorded (under 457 nm excitation) on the glazed surface of the different artifacts (Budai 1, lion, cup, Budai 2, fishes: P599, P600, P857, and P897) of Figure 1. (**a**,**b**): full spectral range; (**a’**,**b’**): zoom on the spectral range presenting the Raman signature of the glassy phases (SiO_4_ bending and stretching modes).

**Figure 9:**
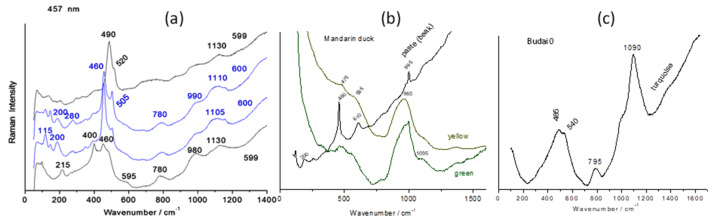
Representative spectra recorded under 457 nm excitation (**a**) on the surface of the body close to the glaze for P600 and P599 fishes; (**b**) on the paste, green, and yellow areas of mandarin duck figurine, (**c**) turquoise Budai 0; peak wavenumber are given.

**Figure 10:**
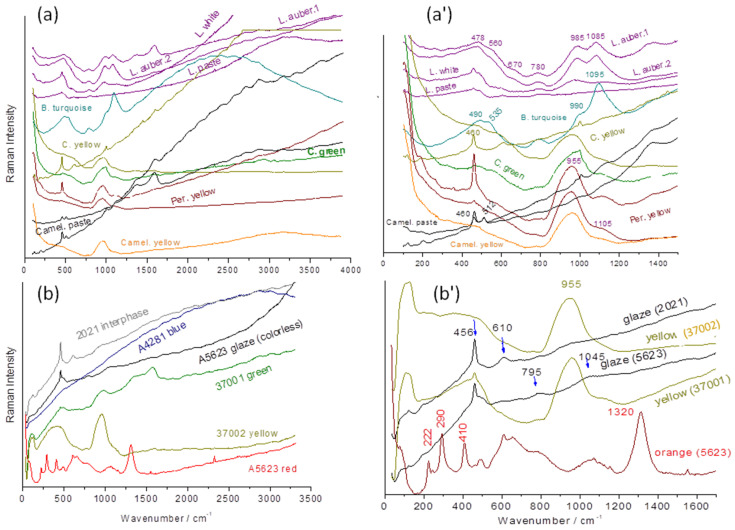
Comparison of the Raman spectra recorded on the glazed surfaces of the selected artifacts (**a**,**a’**) of Figure 1 (L: fo dog; C: (camel yellow) horse; Per: parrot; B: maggot (Budai)) and Figure 2 (**b**,**b’**). On the left (**a**,**b**) exists a full range of spectra and on the right (**a’**,**b’**) spectra within the range of fundamental Raman modes. See Supplementary Information for detailed Raman spectra.

**Figure 11:**
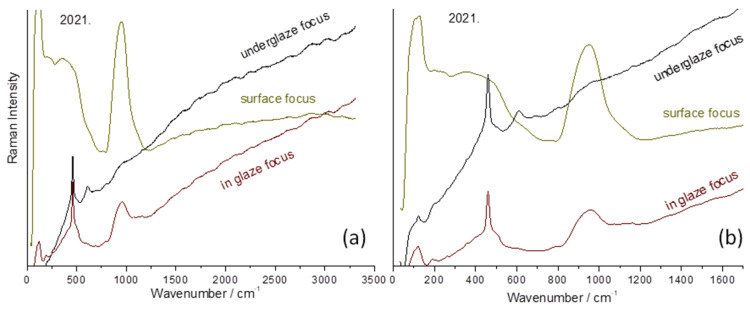
Representative Raman spectra of a bottle (2021.54.1.1-3) recorded by focusing the laser spot from the top surface to the paste–glaze interface using a 200× microscope objective: (**a**) full spectral range; (**b**) zoom.

Figure 8, Figure 9, Figure 10 and Figure 11 compare the Raman signatures recorded over a wide spectral range (in the laboratory) in order to collect both the Raman spectra (the fundamental vibration modes observable for the expected inorganic phases are located between 10 and 1700 cm^−1^) and the luminescence contributions that may exist over the entire recorded domain and beyond [44]. If the fundamental modes make it possible to characterize the crystalline and amorphous phases, the number of modes and their position depending on the composition and the structure (symmetry) of the phases and their width of the structural defects, the luminescence bands (also called fluorescence) generally depend on the presence of certain impurities (transition metals and rare earths) or electronic defects resulting from the use of certain raw materials, preparation, and firing conditions [42,43,44]. The set of spectral contributions can be hence a fingerprint of a kiln production. 

The small difference observed in pXRF between the bodies of P600 and the other objects, for example, P599 in Figure 4, is also evident for the Raman signatures (Figure 8 and Figure 9). The spectra of the P600 body (Figure 9) show the contribution of quartz grains (main peak at ~460 cm^−1^) and feldspar (main peak at ~505 cm^−1^) superimposed on the broad bands around 460, 780, and 1100 cm^−1^ of the vitreous phase typical of porcelain [21,42,49,64]. Fish P599 spectra also show the characteristic doublet of cristobalite (215 and 400 cm^−1^) [64,65] and in certain places (black eyes) the peak at 490 cm^−1^ of the amber chromophore (Fe-S complex) [66] due to (local) iron enrichment. The broad component around 900 cm^−1^ is typical of alkaline glasses containing a small addition of lead [67,68]. The lowest position of the wavenumber is observed for Budai 2 (990 cm^−1^). In agreement with a higher lead content (Figure 5a), the highest (1000 cm^−1^) one is observed for P600 (according its low Pb XRF peak intensity). For Budai 2 and lion, we also note that the bending component at 535 cm^−1^ is better differentiated. The peculiarity observed in the comparison of XRF signals are also found in the Raman analysis. If the thickness of the glaze is too thin, the contribution of the paste modifies the spectrum; for example, for Budai 1 the background is increased. 

The differences between the spectra of the bodies also concern the relative intensities of the bands of the bending modes (around 500 cm^−1^) and stretching (around 1000 cm^−1^) of the vibrational unit of the silicate, the SiO_4_ tetrahedron [69]. The band, with several components of the stretching region, is much more intense than that for the spectra of the bodies, according a less polymerized silicate network, i.e., a glass with a lower melting temperature. The intensity is quite comparable to that of the bending band. Indeed, it was shown empirically [67,68] and by modeling [70] that the ratio of the intensity of these bands was linked to the degree of polymerization of the network of SiO_4_ tetrahedrons and thus the melting temperature of the silicates [67]. High depolymerized silicate networks (i.e., with a high level of flux) exhibit higher intensity of the stretching band. The signatures all present a doublet around 980 and 1090 cm^−1^ for the SiO_4_ stretching modes and another around 480–535 cm^−1^ for the bending modes with small variations. Artifact P600 shows a relatively strong contribution coming from the quartz signature.

The spectra presented in Figure 10 refer to the objects shown in Figure 2, which were analyzed with a mobile Raman spectrometer equipped with a green laser (532 nm). In addition, three other types were observed. We will first focus our attention on the spectral region between 700 and 1300 cm^−1^, where the symmetrical stretching modes of the SiO_4_ tetrahedron are located, namely the structural and vibrational unit of silicate glasses [66,67,68,69,70]. We recognize the signatures (980–1090 cm^−1^ doublet with the more intense component at 1090 cm^−1^) similar to those presented in Figure 8. We distinguish four types of signatures as follows:

- A ~1045 cm^−1^ band with very low intensity;
- A rather strong 1080–1090 cm^−1^ peak with a ca. 980 cm^−1^ shoulder;
- A 980–1000 cm^−1^ doublet of nearly equal intensity;
- An intense single band at ~950 cm^−1^.

The first signature is that of glazes fired simultaneously with the porcelain paste at high temperature (≥1300 °C) [64]. These spectra are not very intense due to the high polymerization degree of the aluminosilicate network [67,68,71,72]. The composition of these high-temperature glazes being close to that of the vitreous phase of the porcelain paste, both formed Al-O and Si-O bonds with a much more ionic character than in lead-containing glasses, hence the low Raman intensity. The narrow peak around 460 cm^−1^ corresponds to the deformation mode of SiO_4_ in the quartz phase, with certain grains of sand not having reacted completely during firing [64].

The Raman signature characterized by a strong ca. 1090 cm^−1^ peak is typical of turquoise glaze.

The doublet of 980–1000 cm^−1^ with equal intensity is observed for the eggplant-colored lion (lead-based glaze deposited on an already glazed body). This signature is rather similar to those shown in Figure 8, but the higher intensity of the 900 cm^−1^ components refers to a higher lead content. The spectra are rather similar to those recorded on Iznik and Kütahya stonepaste [73,74].

The later signature is typical of a lead-rich glaze [68].

The progressive movement of the laser spot from the surface to the interior of the glaze up to its interphase with the paste, which corresponds to approximately 200 µm, is presented in Figure 11. At the very surface, the spectrum of artefact 2021 (bottle, 2021.54.1.1-3) is typical of a lead-rich glaze. Then, focusing deeper through the glaze up to the paste, the spectrum of the mullite phase is identifiable by the intense band at ~600 cm^−1^ and the weak components around 450, 950–1000, and 1000–1100 cm^−1^ [71]. The intermediate layer shows the contribution of both the body and the glaze.

The use of different raw materials can also be deduced from the consideration of the fluorescence components, which are almost non-existent for glasses rich in lead—with the exception of Budai 0—and intense around 2500 cm^−1^ and beyond, which corresponds to an energy of 525 nm for Pb-free or less rich glazes. We find again the conclusions made by examining pXRF data.

## 4. Discussion

The perfectly non-invasive nature, the possibility of modifying the spot size and the depth of the volume analyzed from the µm^3^ to several hundred µm^3^, and the less restrictive regulation of the use of lasers compared to that of ionizing ray sources provide greater ease of measurement on site with mobile Raman instrumentation. While Raman analysis does not make it possible to formulate the composition of the glassy phases, the choice of the analysis volume and the separation of information concerning the crystalline and amorphous phases from the examination of the spectrum (number, shape, width, polarization, and position of the Raman peaks of the phases) [67,68,69] is a decisive advantage in the study of heterogeneous materials such as glazed decoration with pigments and opacifiers. pXRF analysis provides information on elemental composition but in an anisotropic manner (the depth probed depending on the position in the energy signal and therefore a pXRF measurement by the surface, without knowledge of the thickness of the glaze cannot give the lead content reliably) [52]. On the other hand, the Raman analysis localized in a volume much smaller than the thickness of the enamel is not distorted by the contribution of the paste.

It is now well established that the position of the center of gravity of the stretching and bending bands of the SiO_4_ vibrational unit in amorphous or crystalline silicates [66,67,68,69], and the ratio of the areas of these bands, finely characterize the arrangement of the SiO_4_ tetrahedra in the structure of the glass, particularly their degree of connectivity, known as the polymerization index [67]. Table 4 compares representative spectral parameters and their relation to lead levels. A previous study using the SEM-EDS technique for chemical analysis showed that the Q_3_ component, corresponding to a SiO_4_ tetrahedron sharing three of its oxygen atom with the neighboring one (varying between 1020 and 1090 cm^−1^) in the stretching region, is directly correlated with the amount of lead found in the (transparent/white) glaze. The Q_0_ component is observed at low wavenumber (ca. 850 cm^−1^) but with low intensity. Q_1_ and Q_2_ components are located at higher wavenumbers, usually with higher intensity. Q_2_ and Q_3_ are generally the most intense components. The intensity of the Q_4_ component is generally weak, even in silica-rich compositions [67,68,69]. The lead content can be approximately calculated using the empirical formula (Q_3_ = −1.50(wt% PbO) + 1104) determined for rich or poor lead–alkali glazes [73]. The calculated PbO contents are given in Table 4.

For the different types of Raman spectra obtained, the wavenumber of the Q_3_ component almost corresponds to the position of the observed maximum [73]. We observe that the values obtained with the Raman-wt% PbO correlation are higher than those obtained by pXRF. This is understandable because Raman and chemical analysis with EDS are performed by focusing on the surface of the glaze, while the X-ray beam used in pXRF probes both the surface and variable depths, including the lead-free porcelain paste, which distorts the measurement. However, as evidenced by recent reviews on the use of pXRF in the field of Cultural Heritage studies [75,76,77,78,79,80,81], this technique, despite its imperfections, is widely used.

**Figure 12:**
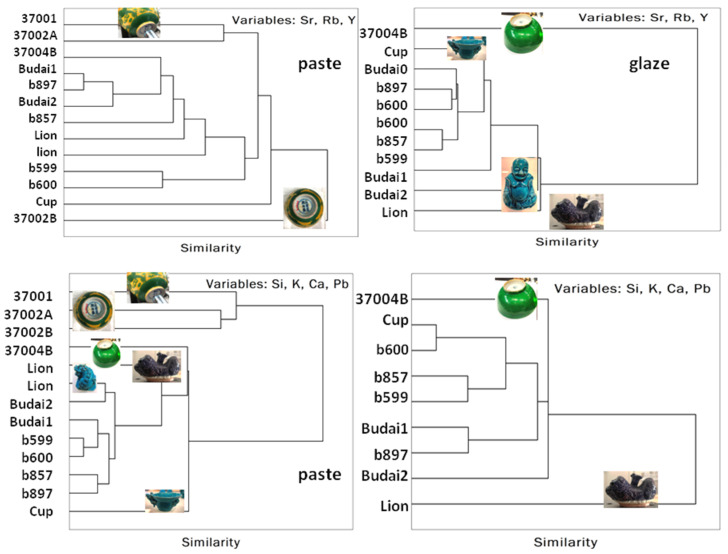
Euclidian hierarchical classification achieved with the trace (Rb, Sr, And Y) and major elements (Si, K, Ca, and Pb) present in the composition of paste and glaze.

Classifications are summarized in Table 5 and hierarchical Euclidean clustering is shown in Figure 12.

The hierarchical classification based on major elements effectively distinguishes between the main types of paste, with the exception that the surface contamination of the green bowl 37004B with lead links its paste to that of other bisques covered with lead-based glaze. Classification based on impurities is more influenced by the use of common or similar raw materials. Regarding the enamels (not all of which were measured using pXRF), the distinct character of the lion, whose eggplant lead enamel is placed on a glaze, stands out clearly, as does the difference observed between Budai.

## 5. Conclusions

Raman is a fast and handy non-invasive technique for the quick classification of the glaze types of scientifically studied ceramic artifacts. Complementary information can be provided with pXRF to identify the use of the same raw materials through an elementary fingerprint. As pointed out in this work, similar visual features may correspond to different production technologies and unexpected compositions. The measurements show that the turquoise monochromes use a category of enamel not listed in the literature, characterized by a slight addition of PbO, close to or lower than 10 wt%, which is low enough not to alter the vivid turquoise color (a higher content would change the color towards jade) but useful for improving the quality of the enamel surface and its shine. This study on the production of Chinese porcelains confirms the efficiency of non-invasive Raman categorization, using a mobile set-up on different types of glazes, as was already demonstrated for other productions from the western part of Asia (Ottoman Empire, Persia, Mongolia) [82]. This work also highlights the difficulty, or even the impossibility, of determining the exact composition of glazes containing lead by pXRF when the thickness of the glaze is less than the depth explored by the photons of the main transitions of the element Pb. Furthermore, the use of borax in Chinese recipes involves that boron and lithium (natural borax contains lithium and sodium) may be also present that make compositions calculated from pXRF data not representatives of the true composition [83]. However, lead-based glazes deposited on an already glazed porcelain can be distinguished from lead-based glazes deposited on the fired body (biscuit) with both techniques.

## Figures and Tables

**Table 1:** Color areas studied for the different artifacts using portable XRF (pXRF), mobile (mRaman), or fixed Raman set-ups.

Artifact	Analyzed Color	Reign	Method	Collection
Buddhist lion figurine	Eggplant White	Kangxi	Raman	Private
Yellow horse figurine	Honey	Kangxi	Raman	
Budai (fo) maggot #0	Turquoise	Kangxi	Raman	
Budai (fo) maggot #1	Turquoise	Kangxi	pXRF, Raman	
Budai (fo) maggot #2	Turquoise	Kangxi	pXRF, Raman	
Mandarin duck figurine	Eggplant	Kangxi	Raman	
	Green		Raman	
	Honey		Raman	
Parrot figurine	Green	Kangxi	Raman	
	Honey			
Libation cup P873a	Turquoise	Kangxi	pXRF, Raman	
Lion P984 figurine	Turquoise	Kangxi	pXRF, Raman	
Fish P857 figurine	Turquoise	Kangxi	pXRF, Raman	
Fish P599 figurine	Turquoise	Kangxi	pXRF, Raman	
Fish P897 figurine	Turquoise	Kangxi	pXRF, Raman	
	Black eyes	Kangxi	Raman	
Fish P600 figurine	Turquoise	Kangxi	pXRF, Raman	
Bowl A4281	Blue	Qianlong?	mRaman	Musée des arts décoratifs, Paris
Bowl 37004B	Green	Qianlong?	pXRF, mRaman	
Bowl 37002A	Green Yellow	Kangxi	pXRF, mRaman	
Bowl 37002B	Green Yellow	Kangxi	pXRF, mRaman	
Bowl 37001	Green Yellow	Kangxi	pXRF, mRaman	
Bowl 5623	Orange-red	Yongzheng?	mRaman	
Budai maggot 16407	Honey Colorless	Kangxi	mRaman	
Bottle 2021.54.1.1-3	Honey	Kangxi?	mRaman	

**Table 2:** Representative compositions (wt%) of yellow, red, and green colored areas from the literature for porcelain with “monochrome” décor (values in italics and bracket correspond to oxides in brackets).

Reign	Color	SiO_2_	Al_2_O_3_	MgO	Na_2_O	K_2_O	CaO	PbO	Fe_2_O_3_ *[FeO]*	P_2_O_5_	TiO_2_	CuO *[Cu_2_O]*	Refs.
Xuande *	yellow	44.9	5.1	0.3	0.85	0.7	0.7	45.9	1.35		0.05		Hou et al., 2022 [60]
Hongzhi *		41.8	5.2	0.3	0.85	0.8	0.7	48.4	1.8		0.05		
Jingdezhen ^+^	yellow	0.7	4.4	0.3	0.7	0.5	0.2	47.9	1.6		0.1		
Ming	turquoise	51.1	0.9	0.3	1.0	7.4	5.0	32.5	0.2			2.2	Wood [61]
Chenghua-5	red	57.5	9.7			6.6	3.7	20.4	1.3	0.5			Cooper et al., 2021 [62]
Qing-1	red	31.5	4.7			1	3.3	55	3.6	0.4			
Qing-3	red	43.2	7			0.75	5.8	38.9	2	-			
Chenghua-1	yellow	34.3	3.45			0.4	0.5	57.3	2.5	1.4			
Qing-1	yellow	47.1	1.5			0.4	0.3	60.3	0.05	0.4			
Chenghua-1	green	44.4	5.2			0.5	0.7	46.2	0.5	1		1.4	
Qing-1		33.4	1.6			0.25	0.4	61.4	0.2	0.4		2.3	
Qing	red	64	14.9	0.4	2.9	2.7	12.7		*[1.7]*			*[0.6]*	Barber et al., 1992 [63]

* Porcelain; ^+^ tile.

**Table 3:** Compositions (wt%) of body, turquoise, yellow, and green areas for some artifacts issued from the instrument software calculation (n.m.: not measured).

Artifact	SiO_2_	Al_2_O_3_	Na_2_O	K_2_O	Li_2_O	B_2_O_3_	CaO	PbO	Fe_2_O_3_	CuO
Lion P984 turquoise glaze	78.01	1.05	n.m.	5.87	n.m.	n.m.	1.46	2.55	0.42	1.19
Budai 1 turquoise glaze	75.52	16.07	n.m.	9.31	n.m.	n.m.	0.66	0.87	0.43	0.13
Budai 2 turquoise glaze	71.98	14.96	n.m.	9.36	n.m.	n.m.	2.13	1.04	0.48	0.05
Bowl 37004B paste	68.71	25.72	n.m.	0.01	n.m.	n.m.	1.82	3.64	0.10	0.01
Bowl 37002B yellow glaze	41.98	12.93	n.m.	0.01	n.m.	n.m.	0.01	45.05	0.01	0.01
Bowl 37002B green glaze	39.81	15.07	n.m.	0.01	n.m.	n.m.	0.01	44.05	0.01	1.05

**Table 4:** Raman classification of the different types of glazes.

Glaze Type	Sample	Main Stretching Mode Component(s) [Shoulder] (cm^−1^)	Main Bending Mode Component(s) (cm^−1^)	Polymerization Index ^+^	Q_3_ (cm^−1^)	PbO Raman ^++^ wt%	PbO XRF ^+++^ wt%
Lead-rich	37002B	955	460	<1	955	>60	45
Lead-rich	2021	955	465	<1	955	>60	n.m.
Lead-poor	P599	[995]1085	480	~1	1085	~13	3
Lead-doped	Lion	990–1090	480–535	~1	1090	~10	2.5
Lead-doped	Budai 2	995–1090	480–535	>1	1092	~8	~1

^+^ ratio of bending to stretching band area after [67]; ^++^ calculated using relationship of [73]; ^+++^ measured by XRF.

**Table 5:** Raman classification of the different types of glazes.

Artifact	View	Analyzed Color	Reign	Main Stretching νSiO_4_ (cm^−1^)	Glaze Type
Buddhist lion figurine (also called *fo* dog)	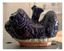	Eggplant White	Kangxi	985/1,085,985/1045	Pb-poor alkaline
Yellow horse figurine	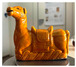	Honey	Kangxi	950	Pb-rich
Budai (fo) maggot #0	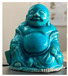	Turquoise	Kangxi	1090	Pb-doped alkaline
Budai (fo) maggot #1	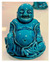	Turquoise	Kangxi	1090	Pb-doped alkaline
Budai (fo) maggot #2	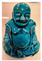	Turquoise	Kangxi	1090	Pb-doped alkaline
Mandarin duck figurine	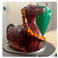	Green	Kangxi	960	Pb-rich
		Honey		960	Pb-rich
Parrot figurine	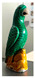	Green	Kangxi	955	Pb-rich
		Honey		960	Pb-rich
Libation cup P873a	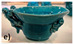	Turquoise	Kangxi	1090	Pb-doped alkaline
Lion P984 figurine	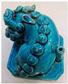	Turquoise	Kangxi	1090	Pb-doped alkaline
Fish P857 figurine	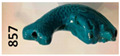	Turquoise	Kangxi	1085	Pb-doped alkaline
Fish P599 figurine	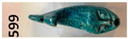	Turquoise	Kangxi	1085	Pb-doped alkaline
Fish P897 figurine	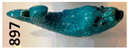	Turquoise	Kangxi	1085	Pb-doped alkaline
Fish P600 figurine	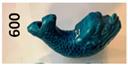	Turquoise	Kangxi	1085	Pb-doped alkaline
Bowl A4281	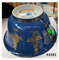	Blue	Qianlong	1040 +	HT alkaline
Bowl 37004B	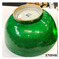	Green	Qianlong	960	Pb-rich
Bowl 37002A	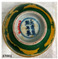	Green Yellow	Kangxi	955 955	Pb-rich Pb-rich
Bowl 37002B	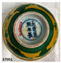	Green Yellow	Kangxi	955 955	Pb-rich Pb-rich
Bowl 5623	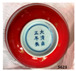	Orange-red	Yongzheng	955	Pb-rich
Bowl 37001	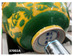	Honey Colorless	Qianlong	960 1045	Pb-rich HT alkaline
Budai maggot 16407	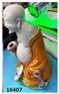	Honey	Kangxi	960	Pb-rich
Bottle 2021.54.1.1-3	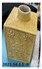	Honey	Kangxi?	955	Pb-rich

+ very poor intensity.

## Data Availability

The original contributions presented in the study are included in the article/Supplementary Material, further inquiries can be directed to the corresponding author.

## Supplementary Materials

The following supporting information can be downloaded at https://www.mdpi.com/article/10.3390/ma17143566/s1, Figure S1: Comparison of the pXRF spectra of some artifacts. Figures S2–S11: As-recorded spectra.
