# Supplementary material for: Non-Invasive Raman Classification Comparison with pXRF of Monochrome and Related Qing Porcelains: Lead-Rich-, Lead-Poor-, and Alkali-Based Glazes
Source: Materials (Basel). 2024 Jul 18;17(14):3566. doi: 10.3390/ma17143566 (PMC11278898; doi:10.3390/ma17143566)
Supplement: Supplementary file 1 [file materials-17-03566-s001.zip › materials-3068471-supplementary.pdf]

Supplementary Information

Non-Invasive Raman Classification Comparison with pXRF of Monochrome and Related Qing Porcelains: Lead-Rich-, Lead-Poor-, and Alkali-Based Glazes

Philippe Colomban [1,*], Xavier Gallet [2], Gulsu Simsek Franci [3], Nicolas Fournery [4], Béatrice Quette [5]

[1] Sorbonne Université, CNRS, MONARIS UMR8233, Campus P. et M. Curie, 4 Place Jussieu, 75005 Paris, France; philippe.colomban@sorbonne-universite.fr

[2] Musée National d'Histoire Naturelle, CNRS, Université Perpignan Via Domitia, Musée de l'Homme, UMR 7194—Histoire Naturelle de l'Homme Préhistorique (HNHP), 17 Place du Trocadéro, 75116 Paris, France; xavier.gallet@mnhn.fr

[3] Koç University Surface Science and Technology Center (KUYTAM), Rumelifeneri Yolu, Sariyer 34450 Istanbul, Türkiye; gusimsek@ku.edu.tr

[4] Galerie Nicolas Fournery, 75001 Paris, France; nf@galerienicolasfournery.fr

[5] Musée des arts décoratifs, 111 rue de Rivoli, 75001, Paris, France; beatrice.quette@madparis.fr

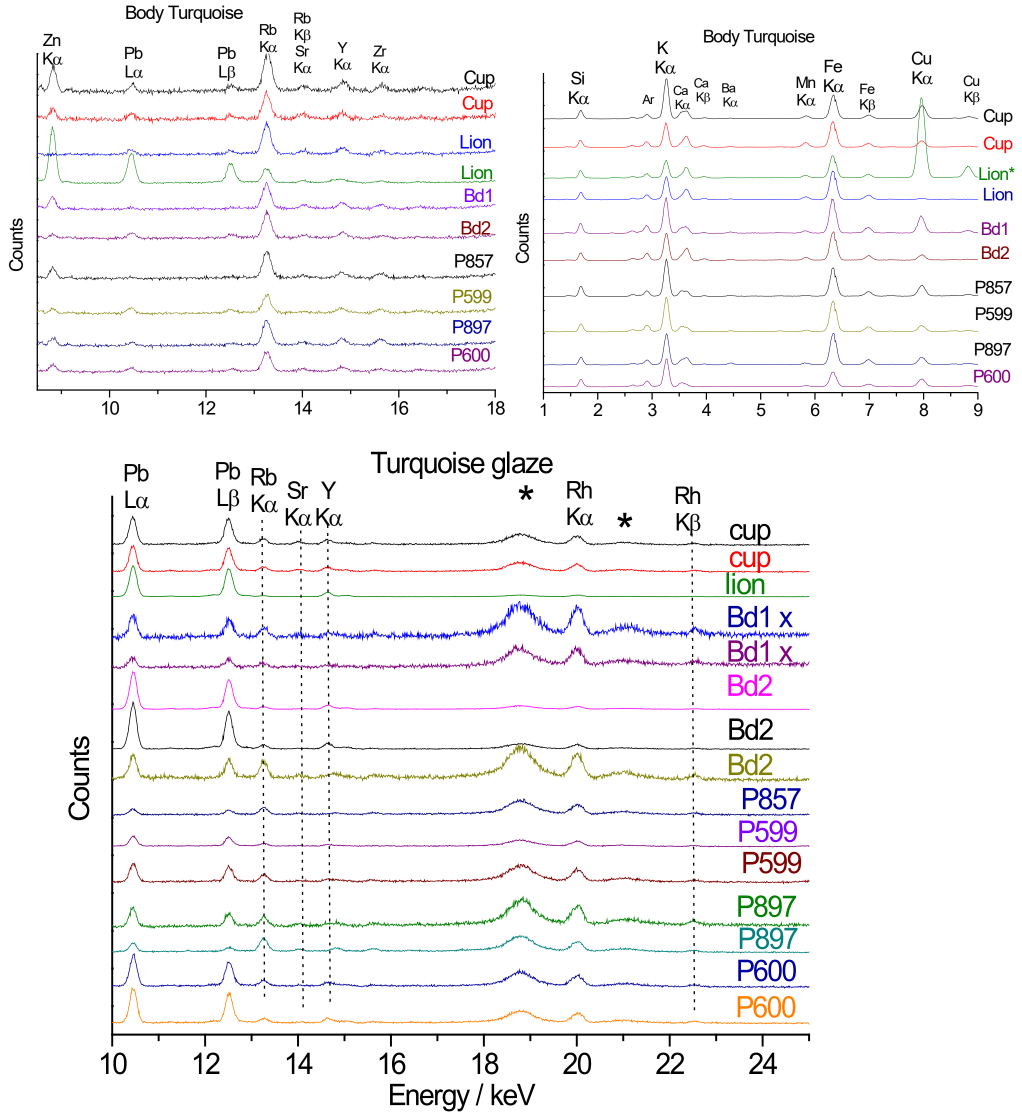

Figure S1. Comparison of the pXRF spectra of some artifacts.

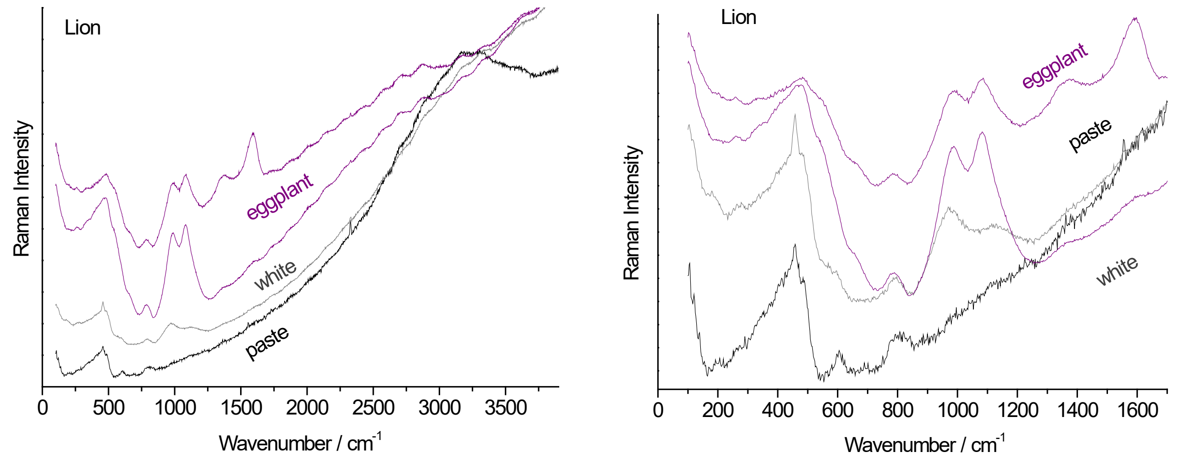

Figure S2. Representative Raman spectra recorded on Lion figurine paste, white/colorless and egg-plant glaze.

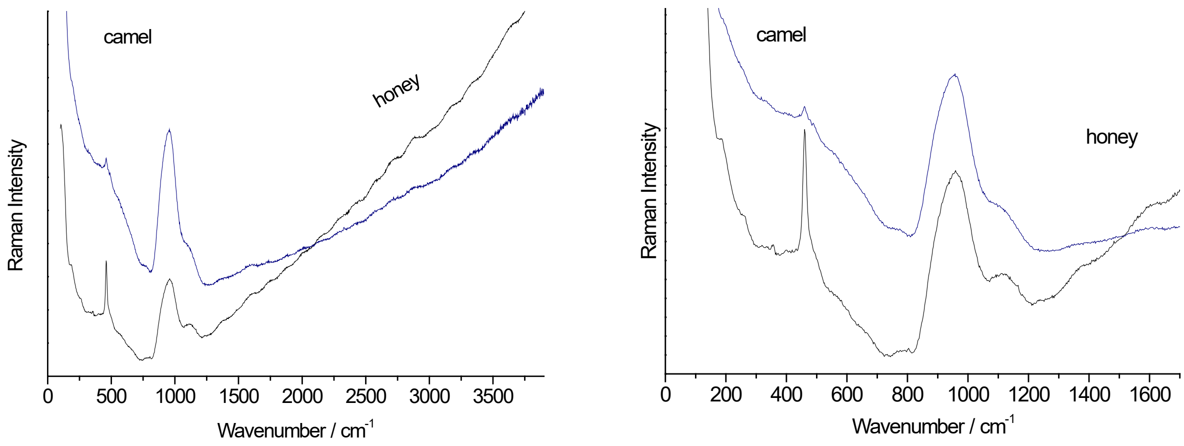

Figure S3. Representative Raman spectra recorded on honey yellow Camel figurine.

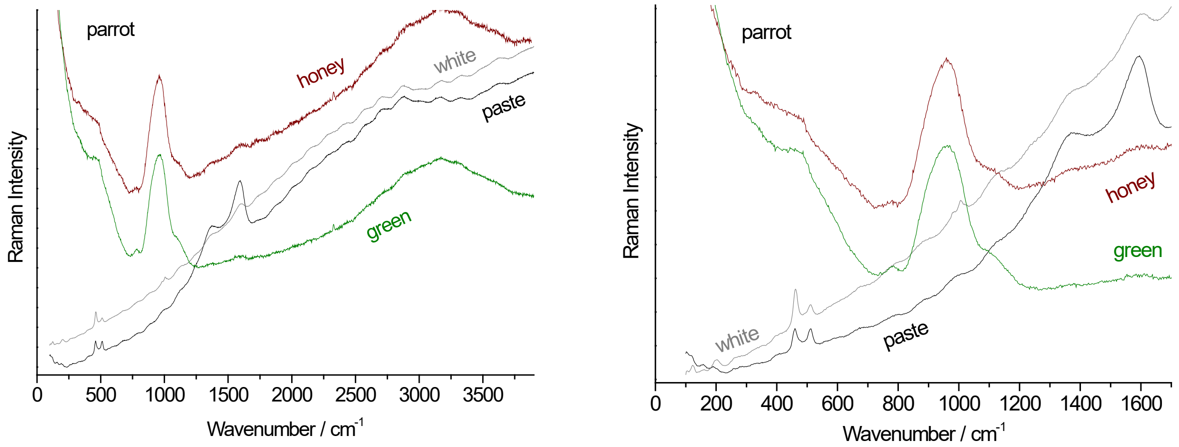

Figure S4. Representative Raman spectra recorded on Parrot figurine paste, white beak, honey and green glaze.

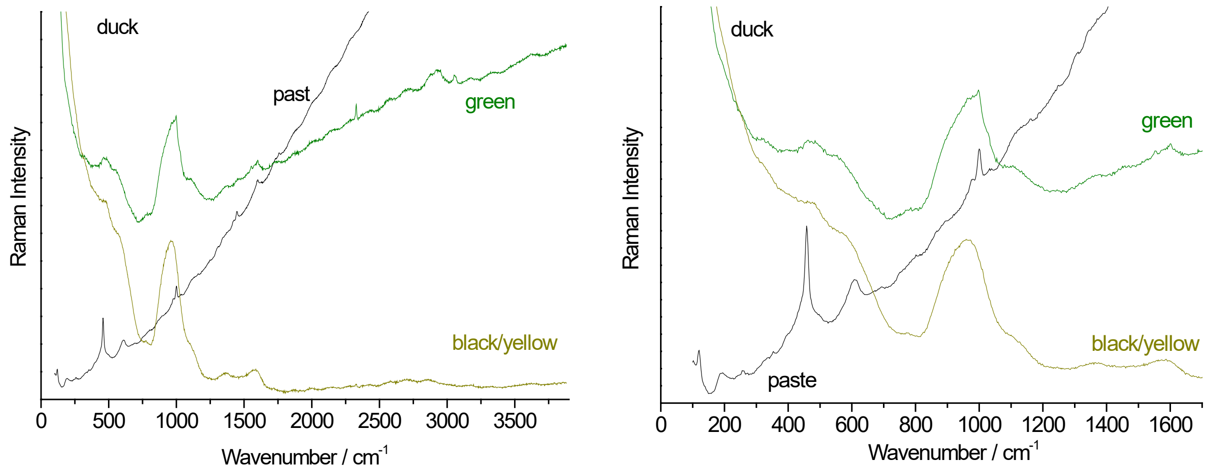

Figure S5. Representative Raman spectra recorded on Duck figurine paste, green and black/yellow glaze.

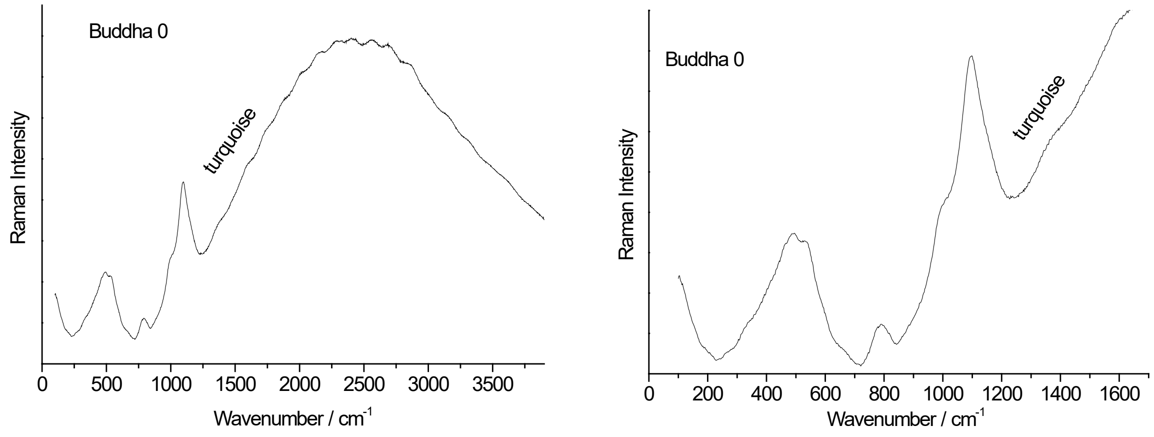

Figure S6. Representative Raman spectra recorded on turquoise Buddha maggot 0 glaze.

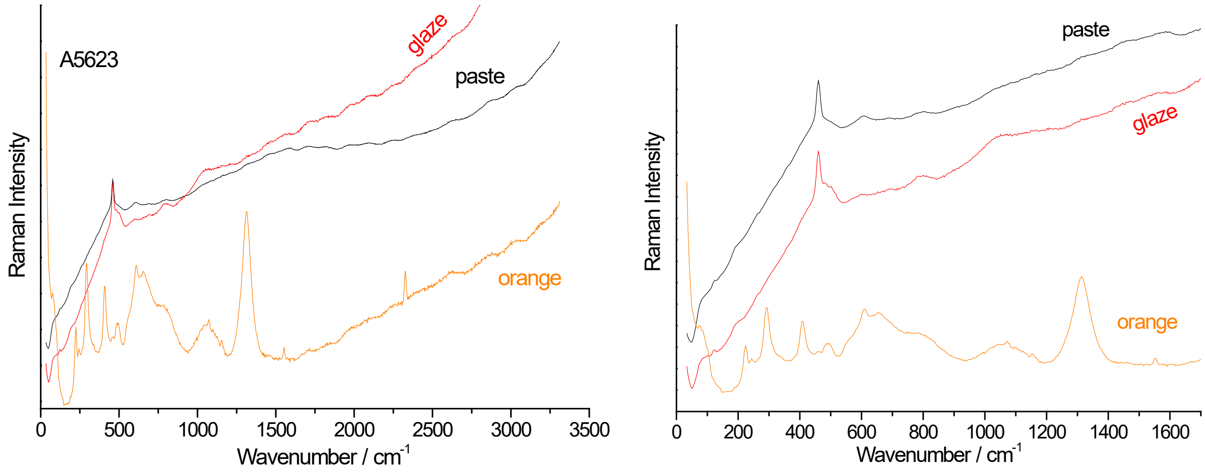

Figure S7. Representative Raman spectra recorded on A5623 bowl paste, colorless and orange-red glaze.

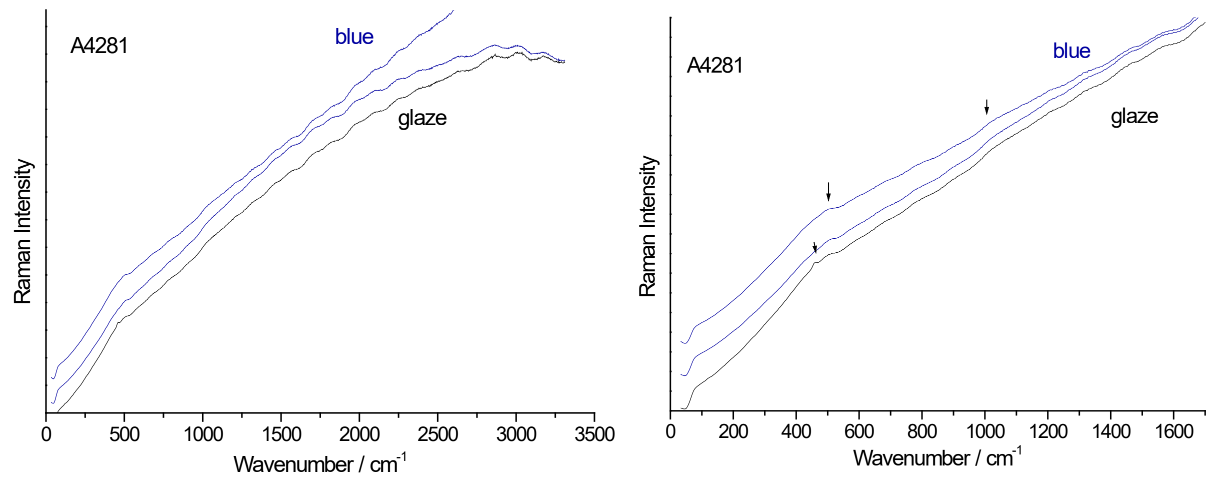

Figure S8. Representative Raman spectra recorded on A4281 bowl colorless and blue glaze.

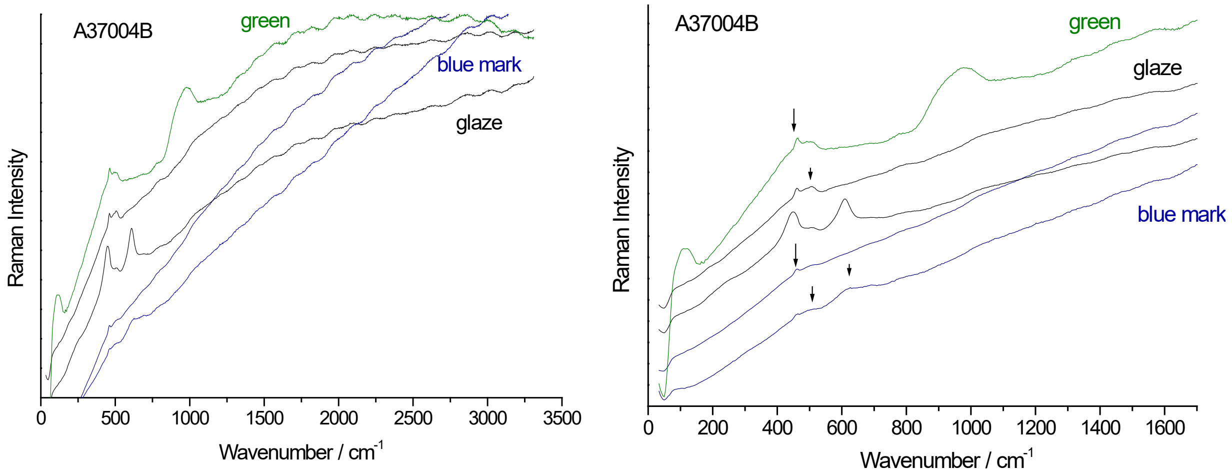

Figure S9. Representative Raman spectra recorded on A37004B bowl colorless, green and blue glaze and blue mark.

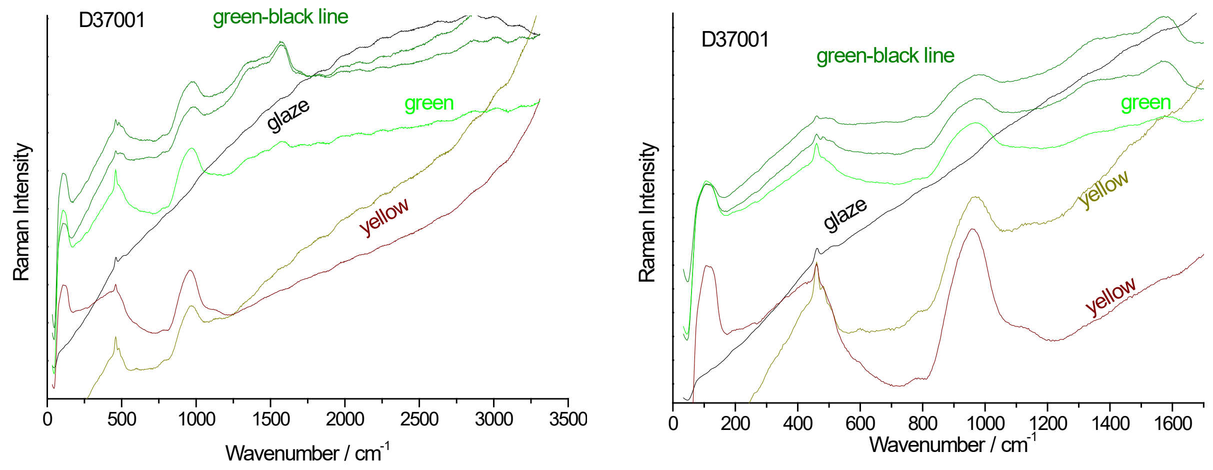

Figure S10. Representative Raman spectra recorded on D37001 bowl colorless, green, honey-yellow glaze and green-black line.

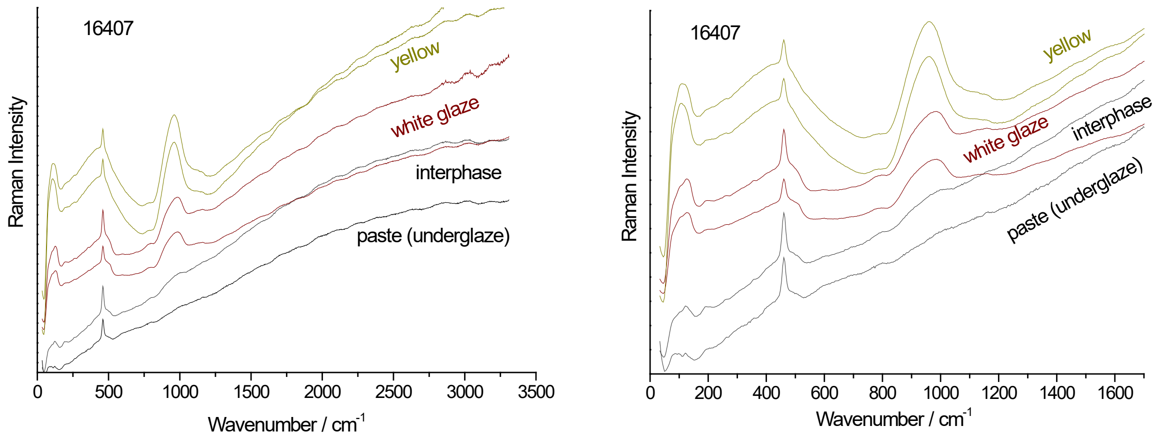

Figure S11. Representative Raman spectra recorded on 16407 Buddha maggot paste, honey-yellow and white glaze, as well as interphase.
